# A novel 4-aminoquinoline chemotype with multistage antimalarial activity and lack of cross-resistance with PfCRT and PfMDR1 mutants

**DOI:** 10.1371/journal.ppat.1012627

**Published:** 2024-10-29

**Authors:** Letícia Tiburcio Ferreira, Gustavo Capatti Cassiano, Luis Carlos Salazar Alvarez, John Okombo, Juliana Calit, Diana Fontinha, Eva Gil-Iturbe, Rachael Coyle, Carolina Horta Andrade, Per Sunnerhagen, Daniel Youssef Bargieri, Miguel Prudêncio, Matthias Quick, Pedro V. Cravo, Marcus C. S. Lee, David A. Fidock, Fabio Trindade Maranhão Costa

**Affiliations:** 1 Laboratory of Tropical Diseases-Prof. Dr. Luiz Jacintho da Silva, Department of Genetics, Evolution, Microbiology and Immunology, University of Campinas-UNICAMP, Campinas, São Paulo, Brazil; 2 Department of Microbiology and Immunology, Columbia University Irving Medical Center, New York, New York, United States of America; 3 Center for Malaria Therapeutics and Antimicrobial Resistance, Columbia University Irving Medical Center, New York, New York, United States of America; 4 Global Health and Tropical Medicine, Associate Laboratory in Translation and Innovation Towards Global Health, LA-REAL, Instituto de Higiene e Medicina Tropical, Universidade NOVA de Lisboa, Lisboa, Portugal; 5 Department of Parasitology, Institute of Biomedical Sciences, University of São Paulo, São Paulo, São Paulo, Brazil; 6 Instituto de Medicina Molecular João Lobo Antunes, Faculdade de Medicina da Universidade de Lisboa, Lisboa, Portugal; 7 Department of Psychiatry, Columbia University Irving Medical Center, New York, New York, United States of America; 8 Wellcome Sanger Institute, Wellcome Genome Campus, Hinxton, United Kingdom; 9 Biological Chemistry and Drug Discovery, Wellcome Centre for Anti-Infectives Research, University of Dundee, Dundee, United Kingdom; 10 Laboratory of Molecular Modeling and Drug Design, Faculty of Pharmacy, Universidade Federal de Goiás, Goiânia, Goiás, Brazil; 11 Center for the Research and Advancement in Fragments and molecular Targets, School of Pharmaceutical Sciences at Ribeirao Preto, University of São Paulo, Ribeirão Preto, São Paulo, Brazil; 12 Center for Excellence in Artificial Intelligence, Institute of Informatics, Universidade Federal de Goiás, Goiânia, Goiás, Brazil; 13 Department of Chemistry and Molecular Biology, University of Gothenburg, Gothenburg, Sweden; 14 Department of Physiology & Cellular Biophysics, Columbia University Irving Medical Center, New York, New York, United States of America; 15 New York State Psychiatric Institute, Area Neuroscience – Molecular Therapeutics, New York, New York, United States of America; 16 Division of Infectious Diseases, Columbia University Irving Medical Center, New York, New York, United States of America; University of South Florida, UNITED STATES OF AMERICA

## Abstract

Artemisinin-based combination therapy (ACT) is the mainstay of effective treatment of *Plasmodium falciparum* malaria. However, the long-term utility of ACTs is imperiled by widespread partial artemisinin resistance in Southeast Asia and its recent emergence in parts of East Africa. This underscores the need to identify chemotypes with new modes of action (MoAs) to circumvent resistance to ACTs. In this study, we characterized the asexual blood stage antiplasmodial activity and resistance mechanisms of LDT-623, a 4-aminoquinoline (4-AQ). We also detected LDT-623 activity against multiple stages (liver schizonts, stage IV-V gametocytes, and ookinetes) of *Plasmodium*’s life cycle, a feature unlike other 4-AQs such as chloroquine (CQ) and piperaquine (PPQ). Using heme fractionation profiling and drug uptake studies in PfCRT-containing proteoliposomes, we observed inhibition of hemozoin formation and PfCRT-mediated transport, which constitute characteristic features of 4-AQs’ MoA. We also found minimal cross-resistance to LDT-623 in a panel of mutant *pfcrt* or *pfmdr1* lines, but not the PfCRT F145I mutant that is highly resistant to PPQ resistance yet is very unfit. No *P. falciparum* parasites were recovered in an *in vitro* resistance selection study, suggesting a high barrier for resistance to emerge. Finally, a competitive growth assay comprising >50 barcoded parasite lines with mutated resistance mediators or major drug targets found no evidence of cross-resistance. Our findings support further exploration of this promising 4-AQ.

## Author summary

The emergence of artemisinin-resistant malaria parasites, first in Southeast Asia and more recently East Africa, highlights the urgency to identify and optimize chemotypes with antimalarial activity that are not compromised by known resistance mechanisms in clinical settings. Using a computer-aided drug discovery approach, we previously identified a 4-AQ (LDT-611) that was active against *P*. *falciparum* asexual blood-stage (ABS) parasites. Herein, we report the multistage antimalarial activity of a structural analog, a side-chain-modified 4-AQ (LDT-623). This analog shares 4-AQ characteristics, such as PfCRT-mediated transport, as well as inhibition of hemozoin formation, that occurs in the parasite digestive vacuole. Cross-resistance profiling showed that this compound was not compromised by known mutations in the digestive vacuole transporters PfCRT or PfMDR1 or by other gene products that govern resistance to antimalarials under development. Efforts to evolve resistance to LDT-623 *in vitro* yielded parasite populations lacking noticeable phenotypic changes. In conclusion, we report a 4-AQ whose MoA is likely to differ from licensed antimalarials within this chemical class, paving the way for further studies on this chemotype as a therapeutic avenue to treat multidrug-resistant malaria.

## 1. Introduction

Malaria remains a major global health concern, with the World Health Organization (WHO) reporting 249 million cases and 608,000 related deaths across 85 malaria-endemic countries and areas in 2022 [1]. Of the human-infecting *Plasmodium* species, *P*. *falciparum* is the most devastating due to its impact on mortality and morbidity, especially in young children and pregnant women. Currently, artemisinin (ART)-based combination therapies (ACTs) constitute the frontline antimalarial treatment policy [1]. ACTs comprise a rapid-acting yet short-lived ART derivative that eliminates most of the initial parasite load, partnered with a longer-lasting molecule with a distinct MoA to eliminate surviving parasites. The efficacy of ACTs has, however, been compromised by the emergence of mutant *k13* parasites in Southeast Asia [2–4] and parts of East Africa [5–7]. Parasites with specific *k13* mutations exhibit partial resistance to artemisinin, characterized by delayed clearance time upon treatment with an ART derivative or an ACT [8–10] and higher levels of ring-stage parasite survival *in vitro* [11]. This threat to the long-term therapeutic utility of ACTs calls for urgent efforts to identify and develop new molecules, preferably with divergent MoAs, no cross-resistance to existing molecules, and pan-activity across multiple stages of the *Plasmodium* lifecycle.

In the human host, the *P*. *falciparum* lifecycle involves the invasion of hepatocytes by sporozoites injected by a feeding female *Anopheles* mosquito. Liver-stage parasites mature and are released into the bloodstream as merozoites that invade red blood cells (RBCs) to initiate the pathogenic asexual blood stage (ABS) cycle. This cycle lasts ~48 hours and is characterized by differentiation of the merozoites into ring forms then into trophozoites and ultimately schizonts, from which more merozoites egress and reinvade other RBCs. Some merozoites can also differentiate into sexual gametocyte forms that once mature can be transmitted to a feeding mosquito.

ABS parasites digest host hemoglobin (Hb) in their acidic digestive vacuole (DV), leading to the release of essential amino acids for intracellular growth and free heme as a by-product. In its labile form, free heme is toxic due to its ability to induce free radical formation. Parasites counter this toxicity by converting heme into inert hemozoin (Hz) crystals [12]. Extensive evidence shows that 4-AQ drugs such as chloroquine (CQ) and piperaquine (PPQ) exert their antiplasmodial activity by accumulating within the DV and binding to heme monomers and dimers, effectively interfering with this detoxification pathway [13]. This antiplasmodial activity is predicated on the structurally unique pharmacophore of these compounds that comprises the 4-amino-7-chloroquinoline nucleus for heme binding and inhibition of Hz formation as well as a tertiary amino group in the side chain for vacuolar drug accumulation [14,15]. However, point mutations in the *P*. *falciparum* chloroquine resistance transporter (PfCRT) [16], an oligopeptide and drug transporter located on the DV membrane [17], have been identified as the main driver of resistance to CQ, PPQ, and some related quinolines [18]. Polymorphic PfCRT variants carry 4 to 10 mutations that can differentially modify parasite susceptibility to CQ and PPQ [19,20]. However, several 4-AQs maintain similar activity against both CQ-sensitive and CQ-resistant parasites, suggesting that they are not recognized and exported by mutant PfCRT [21]. This has informed the introduction of more versatile and potent 4-AQs like amodiaquine, pyronaridine, and ferroquine in clinical use as effective alternatives to address resistance [22].

Herein, we identify the multistage antiplasmodial activity of LDT-623. This compound is a side-chain-modified 4-AQ structurally analogous to LDT-611 that was previously identified in a screening campaign on a library of ~120,000 natural products and derivatives for potential antiplasmodial activity [23]. To evaluate the activity of LDT-623 against other stages of the *P*. *falciparum* lifecycle, we tested its gametocytocidal potential and ability to inhibit other exoerythrocytic forms of the parasite. Furthermore, we investigated its MoA and possible cross-resistance with known drug targets. Our results open up the possibility of further exploring this compound as a potential antimalarial.

## 2. Material and methods

### 2.1. Compound preparation

Screening compounds were purchased from providers Chembridge, MedChemExpress, InterBioScreen, ChemDiv Inc, and BLD Pharmatech Ltd, through the MolPort website (www.molport.com) and were further dissolved in DMSO in 10 mM stock solutions. Standard antimalarial drugs were purchased from Sigma-Aldrich and prepared as 10 mM stocks.

### 2.2. Virtual screening workflow for the identification of LDT-611 structural analog molecules

Based on the premise that structurally similar compounds may possess similar biological activity, we carried out a virtual screening of the MolPort library of ~7.5 million compounds to select molecules with relevant structural similarity to LDT-611. We employed the “Find similar” function that utilizes the Tanimoto coefficient, a quantitative measure of chemical scaffold similarity, to filter molecules with structural similarity. A similarity cutoff of 78% (T_c_ = 0.78) was defined based on a visual inspection of analog compounds, to keep those that were 4-AQ and resembled LDT-611’s structure. Further steps removed double entries and molecules whose antimalarial activity had been previously reported on PubChem. A final filter removed acridines from the dataset. Finally, the selected analogs were purchased and evaluated *in vitro*.

### 2.3. *P*. *falciparum in vitro* culture and ABS assays

*P*. *falciparum* ABS parasites were cultured at 3% hematocrit in human O^+^ RBCs in RPMI-1640 media supplemented with 25 mM HEPES, 50 mg/L hypoxanthine, 2 mM L-glutamine, 0.21% sodium bicarbonate, 0.5% (w/v) AlbuMAXII (Invitrogen) and 10 μg/mL gentamycin. Culture flasks were kept in modular incubator chambers (Billups-Rothenberg) in a 5% O_2_, 5% CO_2_, and 90% N_2_ atmosphere at 37°C. Synchronized ring-stage parasites were obtained by two consecutive rounds of treatment with 5% D-sorbitol in 48-hour intervals [24]. ABS parasite survival assays were performed to define half-maximal compound concentrations that inhibit 50% or 90% parasite growth (IC_50_ and IC_90_). This was done by exposing cultures at 1% hematocrit and 0.3% parasitemia in 96-well plates in the presence of drugs in a two-fold 10 or 12-point serial dilution in duplicate along with drug-free controls. After a 72-hour incubation, parasite survival was assessed by flow cytometry on an iQue flow cytometer (Sartorius) using SYBR Green and MitoTracker Deep Red FM (Life Technologies) as nuclear stain and vital dye, respectively. Parasite survival was determined as a percentage relative to drug-free control. IC_50_ values were interpolated from log concentration *versus* response curves in Prism 9 (GraphPad Prism Software Inc.).

### 2.4. Cytotoxicity assays

Human hepatoma (HepG2) and fibroblast-like monkey kidney cells (COS-7) were cultured in Dulbecco’s Modified Eagle Medium supplemented with 10% heat-inactivated fetal bovine serum and gentamycin (40 mg/L) at 37°C in a 5% CO_2_ atmosphere. Cytotoxicity of test compounds was measured by metabolic reduction of mitochondrial enzymes using the MTT assay (3-[4,5-dimethyl-thiazol-2-yl]-2,5-diphenyltetrazolium chloride) [25]. Cells were seeded at 10^5^ cells/well in 96-well plates and incubated for 72 hours in the presence of a 2-fold 12-point serial dilution of drugs along with drug-free controls. After adding 15 μL of MTT (5 mg/mL) to the wells, plates were centrifuged at 317g for 5 minutes and the pellet was resuspended in isopropanol. Absorbance was measured at 570 nm (CLARIOStar, Labtech BMG). Cell viability was determined by formazan production, and was expressed as a percentage of untreated control wells.

### 2.5. *P*. *falciparum* gametocyte production and growth inhibition assay

*P*. *falciparum* NF54-Pfs16-GFP-Luc (MRA-1217), a highly gametocyte-producing strain, was cultured following standard procedures [26]. NF54-Luc ABS parasites were sorbitol-synchronized as previously described [24] and cultured to reach 8–10% parasitemia as mature trophozoites prior to starting gametogenesis induction via nutrient deficit stress [27]. Briefly, stress was induced by replacing only 40% of the culture medium and returning cultures to a 37°C incubation. Cultures were treated with 50 mM N-acetylglucosamine for 10 days to remove ABS parasites. Mature gametocytes (stages IV-V) were isolated using magnetic columns and incubated at 2% gametocytemia and 1.5% hematocrit in 96-well plates containing the test compounds for 48 hours at 37°C, following standard *P*. *falciparum* culturing conditions. Methylene blue was used as a gametocytocidal control alongside drug-free wells. Luciferase activity was assessed by adding 40 μL of luciferin substrate (Promega) to 40 μL of parasite lysates and detecting bioluminescence at an integration constant of 10s with the BioTek Cytation 5 Cell Imaging Multimode Reader [28].

### 2.6. *In vitro* inhibition of *P*. *berghei* ookinete formation

To measure the inhibition of gamete conversion into ookinetes, a mutant luciferase-expressing *P*. *berghei* line (nLuc) was intraperitoneally injected into BALB/c mice [29]. After 3–4 days, gametocyte-carrying blood samples were collected. *In vitro* compound testing was carried out by exposing 4 μL of infected blood mixed with 80 μL of culture medium (RPMI-1640 supplemented with 25 mM HEPES, 50 mg/L hypoxanthine, and 1% penicillin/streptomycin/neomycin at pH 8.3) to a range of drug concentrations for 24 hours at 21°C. Following the incubation, 20 μL of nLuc substrate (Nano-Glo Luciferase Assay System, Promega) was added to each well, and luminescence was measured in a luminometer. Increased light emission correlated with nLuc production and subsequent luciferase activity due to *in vitro* fertilization and ookinete conversion, as luciferase expression in this parasite line is regulated by the CTRP promoter that is ookinete-specific [30]. Conversion inhibition was calculated as the percentage of luciferase activity of drug-exposed wells relative to untreated ones, from which IC_50_ values were interpolated.

### 2.7. *In vitro* inhibition of liver stage infection

*In vitro* inhibition of *Plasmodium* liver stage infection was assessed using bioluminescence data from Huh-7 cells infected with a firefly-luciferase-expressing *P*. *berghei* line, *Pb*GFP-Luc_con_ [31], in the presence of various compound concentrations. Huh-7 cells, a human hepatoma cell line, were cultured in RPMI-1640 medium supplemented with 10% fetal bovine serum, 1% nonessential amino acids, 1% glutamine, 1% penicillin/streptomycin and 10 mM HEPES at pH 7 at 37°C under 5% CO_2_. One day before drug treatment and infection, 10^4^ Huh-7 cells/well were seeded in 96-well plates. The medium was then replaced by an infection medium (culture medium supplemented with 50 μg/mL gentamycin and 0.8 μg/mL amphotericin B) containing desired concentrations of test compounds. Fresh sporozoites recovered from salivary glands of infected female *Anopheles stephensi* mosquitoes were added to seeded Huh-7 cells at a 1:1 proportion, followed by centrifugation at 1700g for 5 minutes and incubation for 46 hours at 37°C under 5% CO_2_. Compound toxicity against Huh-7 cells was assessed by measuring cell confluency using the Alamar Blue assay (Invitrogen). IC_50_ values were calculated using nonlinear regression analysis (GraphPad Prism version 9).

### 2.8. PfCRT transport measurement assays

Purified PfCRT variants were reconstituted in preformed liposomes made of *E*. *coli* total lipids:cholesteryl hemisuccinate 97:3 (w/w) at a protein-to-lipid ratio of 1:150 (w/w). The lumen of the proteoliposomes was composed of 100 mM KPi, pH 7.5, and 2 mM β-mercaptoethanol. Uptake of [^3^H]CQ (1 Ci/mmol) or [^3^H]PPQ (1 Ci/mmol) was performed by diluting PfCRT-containing proteoliposomes (30 ng of PfCRTper reaction) in an uptake buffer containing 100 mM Tris/MES, pH 5.5, in the presence or absence of 1 μM of the test compounds. In addition, 1 μM valinomycin was added to the reaction to generate a K^+^ diffusion potential-driven membrane potential (ΔΨ). Reactions were stopped after 30 seconds by the addition of ice-cold 100 mM KPi, pH 6.0, and 100 mM LiCl, and filtered through 0.45-μm nitrocellulose filters (Millipore). Filters were dried and incubated in a scintillation cocktail, and the radioactivity captured on the filters was counted in a Hidex SL300 scintillation counter. The efficiency of detection was calculated with a standard curve of known concentrations of each radiolabeled compound, and this was used to transform decays per minute (dpm) into pmol. The nonspecific interaction of each compound with nitrocellulose filters was determined by measuring mock uptake in the absence of liposomes or proteoliposomes. These values (determined for each experiment) were used to calculate background uptake in liposomes or proteoliposomes. Drug-specific uptake was determined by subtracting the time-dependent accumulation of the tested compounds in control liposomes (lacking PfCRT) from that measured in PfCRT-containing proteoliposomes [20].

Solid Supported Membrane (SSM)-based electrophysiological measurements were performed using SURFE^2^R N1 technology (Nanion Technologies, Inc.) according to published protocols [32,33]. Briefly, sensors were coated with 1-octadecanethiol (Sigma-Aldrich), rinsed, and 1,2-diphytanoyl-sn-glycero-3-phosphocholine (Avanti Polar Lipids, Inc) was added. Immediately, the sensor was filled with 50 μL non-activating buffer (10 mM Tris-MES, pH 5.5) and PfCRT-containing proteoliposomes (PLs). To generate a K^+^ diffusion potential-driven ΔΨ, valinomycin was included at a final concentration of 80 nM [34,35]. The activating solution (10 mM Tris-MES, pH 5.5 containing either 10 μM CQ, PPQ, LDT-623, pyrimethamine, or atovaquone) was applied using a single-solution exchange protocol (activating buffer incubation for 1 second). Each measurement was repeated at least twice employing at least three different sensors. Peak currents for PfCRT were corrected by subtracting the peak currents recorded with control liposomes (lacking this transporter). To acquire the PfCRT-elicited charge (Coulomb) movement associated with drug transport, the area under the curve (current as a function of time) was analyzed with GraphPad Prism version 10.

### 2.9. Cellular heme fractionation assay

A heme fractionation assay was used to measure the inhibition of Hz formation in cultured ABS *P*. *falciparum* 3D7 parasites as previously described [36]. Briefly, ring-stage 3D7 parasites were treated with consecutive D-sorbitol and Percoll solutions to obtain tightly synchronized 3 hours post-invasion young rings that were incubated with test drugs (0.5–3.0 × IC_50_) alongside a vehicle control in standard culturing conditions (see above). Late trophozoites were harvested after 32 hours of incubation by lysing RBCs with 0.05% saponin followed by multiple washes with 1 × PBS (pH 7.5) to remove any residual traces of hemoglobin. The parasite pellet was resuspended in PBS and the total number of trophozoites isolated was quantified using flow cytometry. Contents of the trophozoite pellet were released by hypotonic lysis and sonication. After centrifugation, treatments with HEPES buffer (pH 7.4), sodium dodecyl sulfate, pyridine, and NaOH, allowed us to separate the fractions corresponding to the different heme species of interest. A microplate reader was employed to measure the UV-visible spectrum of each heme fraction as a Fe(III)heme-pyridine complex. Fractions of undigested hemoglobin, “free” heme, and Hz were quantified using a heme standard curve based on the mass of each heme-Fe species per mature trophozoite (fg/cell), obtained by dividing the total amount of each heme species by the corresponding number of parasites in that fraction as determined by flow cytometry. Statistical comparisons and analyses for trends employed Students’ *t*-tests (GraphPad Prism version 9).

### 2.10. Resistance selection experiments

Resistance to LDT-623 was assessed using single-step *in vitro* resistance selection protocols [37]. Duplicate flasks with 1 × 10^9^ *P*. *falciparum* Dd2-Pol δ parasites were exposed to 3 × IC_50_ under two different drug regimens: (1) removal of drug pressure as soon as live parasites cleared, and (2) drug pressure kept constant throughout the experiment. Dd2-Pol δ is a hypermutable line with two point mutations in DNA polymerase δ that compromise its proofreading activity [38] Cultures were monitored daily by thin blood smears until live parasites were no longer detectable. Drug-containing media was changed daily until parasites cleared, and every other day thereafter. Cultures were passaged once weekly by reducing the volume by 25% and replacing 1/3 of RBCs. Recrudescence monitoring was carried out twice a week by thin blood smears for 60 days. In the event of recrudescence, parasite susceptibility to LDT-623 was tested as described above.

### 2.11. Resistome growth assay

The resistome growth assay employs a parasite pool consisting of 51 barcoded lines including both Dd2 and 3D7 genetic backgrounds, with wild-type lines for both (S3 Table). Most lines used were derived from *in vitro* resistance selection experiments, with others having been CRISPR edited. All lines were barcoded via CRISPR editing of a 101bp barcode cassette into the non-essential *pfpare* locus (PF3D7_0709700) [39]. Parasites were cultured in RPMI-1640 medium supplemented with HEPES (5.94 g/L), NaHCO_3_ (2.1 g/L), glutamax (1×), hypoxanthine (50 mg/L), gentamycin (25 μg/L), and AlbumaxII (5 g/L) at a 50% hematocrit. Parasite growth was monitored over 14 days by flow cytometry (Cytoflex, Beckman Coulter) by staining with 1× SYBR Green and 0.2 μM MitoTracker Deep Red. Parasitemia adjustments were made to maintain a healthy range of <5% parasitemia. Parasite cultures were harvested at day 14 and lysed by saponin (0.05%), with pellets washed twice with PBS. Barcodes were PCR amplified using 5 μL of parasite pellet and sequenced by Illumina amplicon sequencing [40].

## 3. Results

### 3.1. Identification and activity profiling of LDT-611 and its analogs

**Fig 1:**
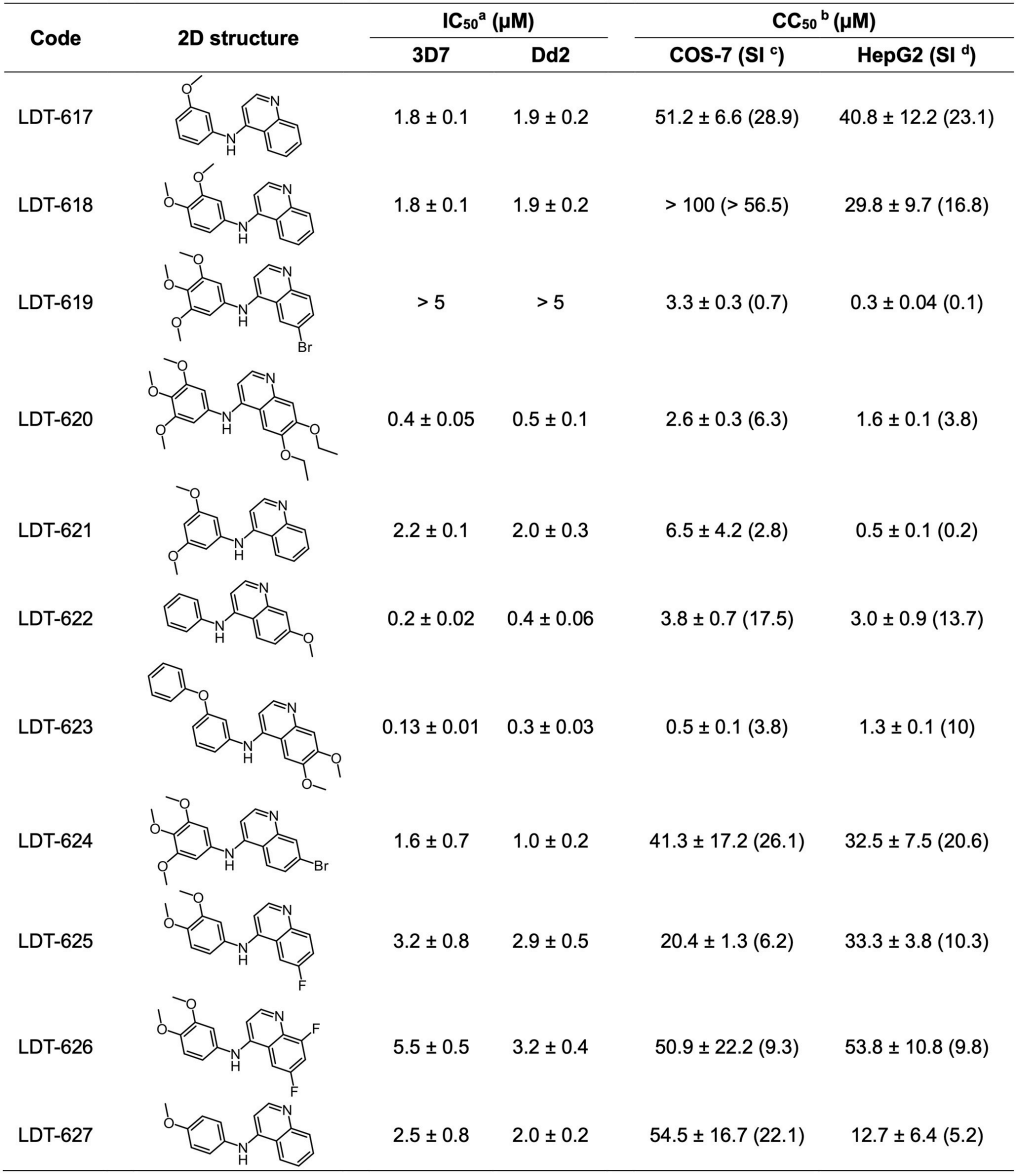
*In vitro* activity of LDT-611 analogs against *P*. *falciparum* asexual blood stages and mammalian cell lines. ^a^ IC_50_: Mean half-maximal inhibitory concentration and their respective standard errors, assayed against *P*. *falciparum* 3D7 and Dd2 ABS parasites (N = 3); ^b^ CC_50_: Mean half-maximal cytotoxic concentration against mammalian cell lines (N = 3); ^c^ SI: Selectivity index was calculated by dividing the CC_50_ (against COS-7) by the IC_50_ (against 3D7); ^d^ SI: Selectivity index was calculated by dividing the CC_50_ (against HepG2) by the IC_50_ (against 3D7). Data are represented by mean ± standard deviation.

In a previous study, we identified several biodiversity-derived compounds with *in vitro* activity against *P*. *falciparum* ABS parasites *in vitro* [23]. To further explore the antimalarial potential of these hits, we chose LDT-611 (S1A Fig) due to the combination of its sub-micromolar activity against ABS parasites, selectivity index > 10, and relatively low structural similarity to clinical antimalarials. LDT-611 inhibited the growth of CQ-sensitive (3D7) and CQ- and mefloquine-resistant (W2) parasite strains with IC_50_ values of 760 and 820 nM, respectively (S1B Fig). Its equipotency against wild-type and mutant PfCRT isoforms suggests that this compound can circumvent CQ resistance. To identify a more potent compound, we used the Tanimoto coefficient as part of an optimization pipeline that yielded 11 available analogs (S1C Fig). Tanimoto coefficients (S1D Fig) showed that at least 78% of their structures were shared (T_c_ = 0.78), with the potent antimalarial 4-AQ nucleus remaining intact. These 11 analogs were then evaluated for their activity against *P*. *falciparum* 3D7 and Dd2 ABS parasites, along with cytotoxicity assays with COS-7 and HepG2 mammalian cell lines (Fig 1).

### 3.2. LDT-623, the most potent analog, exhibits multistage antiplasmodial activity

**Fig 2:**
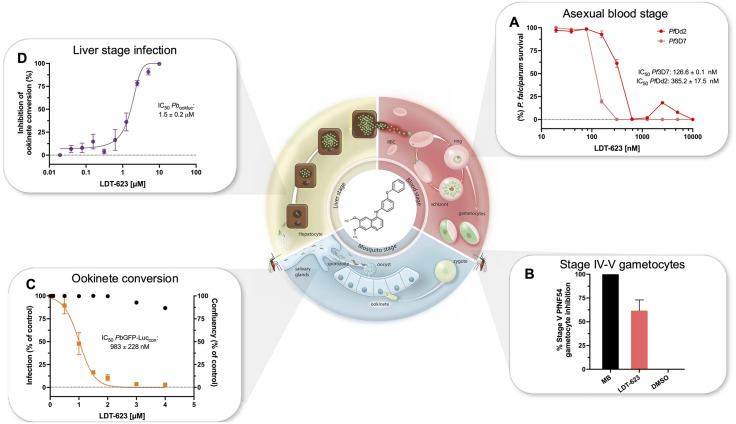
Antiplasmodial activity of LDT-623 throughout the *Plasmodium* lifecycle. (A) LDT-623 *in vitro* activity against ABS *P*. *falciparum* CQ-sensitive 3D7 and CQ-resistant Dd2 strains. Mean IC_50_: 126.6 and 365.2 nM, respectively. Parasite survival was calculated as a percentage of untreated control. Data represent mean ± SEM (N = 2–3 independent assays, with technical duplicates). (B) Percent activity of LDT-623 against late-stage *P*. *falciparum* NF54 gametocytes at 5 μM. Gametocyte viability was assessed via bioluminescence. Data represent mean ± SEM (N = 3). MB: methylene blue; DMSO: dimethyl sulfoxide. (C) Dose-response curve for LDT-623 concentration *versus* inhibition of *P*. *berghei* ookinete formation compared to vehicle-treated control. IC_50_: 1.5 μM. Data represent mean ± SEM (N = 7). (D) Pre-erythrocytic activity of LDT-623. IC_50_: 983 nM. Dose-response curve represents hepatic stage infection as a percentage of drug-free control assessed by luminescence 48h after infection of Huh-7 cells with *P*. *berghei* sporozoites, while black dots express host cell confluency. Data represent mean ± SEM (N = 2).

LDT-623 was the most potent of the analogs tested against ABS parasites, with IC_50_ values of 127 and 365 nM for 3D7 and Dd2 ABS parasites, respectively) (Fig 2A). This compound was therefore selected for further experimental evaluation throughout the *Plasmodium* lifecycle (S1 Table).

The potential transmission-blocking activity of LDT-623 was assessed by exposing NF54 stage IV-V gametocytes to 5 μM of test compounds. LDT-623 inhibited 62% of mature gametocyte viability compared to untreated control (Fig 2B), an unusual activity for 4-AQs [41]. Gametocytocidal activity was assessed alongside methylene blue, a positive control that showed 100% activity at the same 5 μM concentration. We also employed a *P*. *berghei* model to evaluate LDT-623’s inhibition of ookinete formation *in vitro*, using a conversion assay that evaluates the inhibition of gamete fertilization into a zygote and subsequent conversion into an ookinete. LDT-623 yielded an IC_50_ of 1.5 μM (Fig 2C). For reference, CQ inhibited 56.7% of zygote conversion into ookinetes at 10 μM (S2 Fig).

We also observed pre-erythrocytic activity of LDT-623 using an *in vitro* firefly luciferase-expressing *P*. *berghei* line by measuring inhibition of hepatocyte infection based on luminescence intensity [31]. In agreement with its IC_50_ of 983 nM (Fig 2D), LDT-623 showed 52.1% inhibition of hepatic infection at 1 μM. The positive control atovaquone almost completely ablated infection at 10 nM, consistent with its known liver stage activity [42]. Of note, we observed minimal to no dose-dependent toxicity towards Huh-7 host cells, as evidenced by over 86% cell viability at the maximum concentration of 4 μM LDT-623.

### 3.3. LDT-623 inhibits intracellular hemozoin formation

The 4-AQ nucleus, contained by antimalarial drugs such as CQ and PPQ, interferes with the heme detoxification pathway inside the parasite DV through complexation with heme, leading to inhibition of Hz formation [43,44]

**Fig 3:**
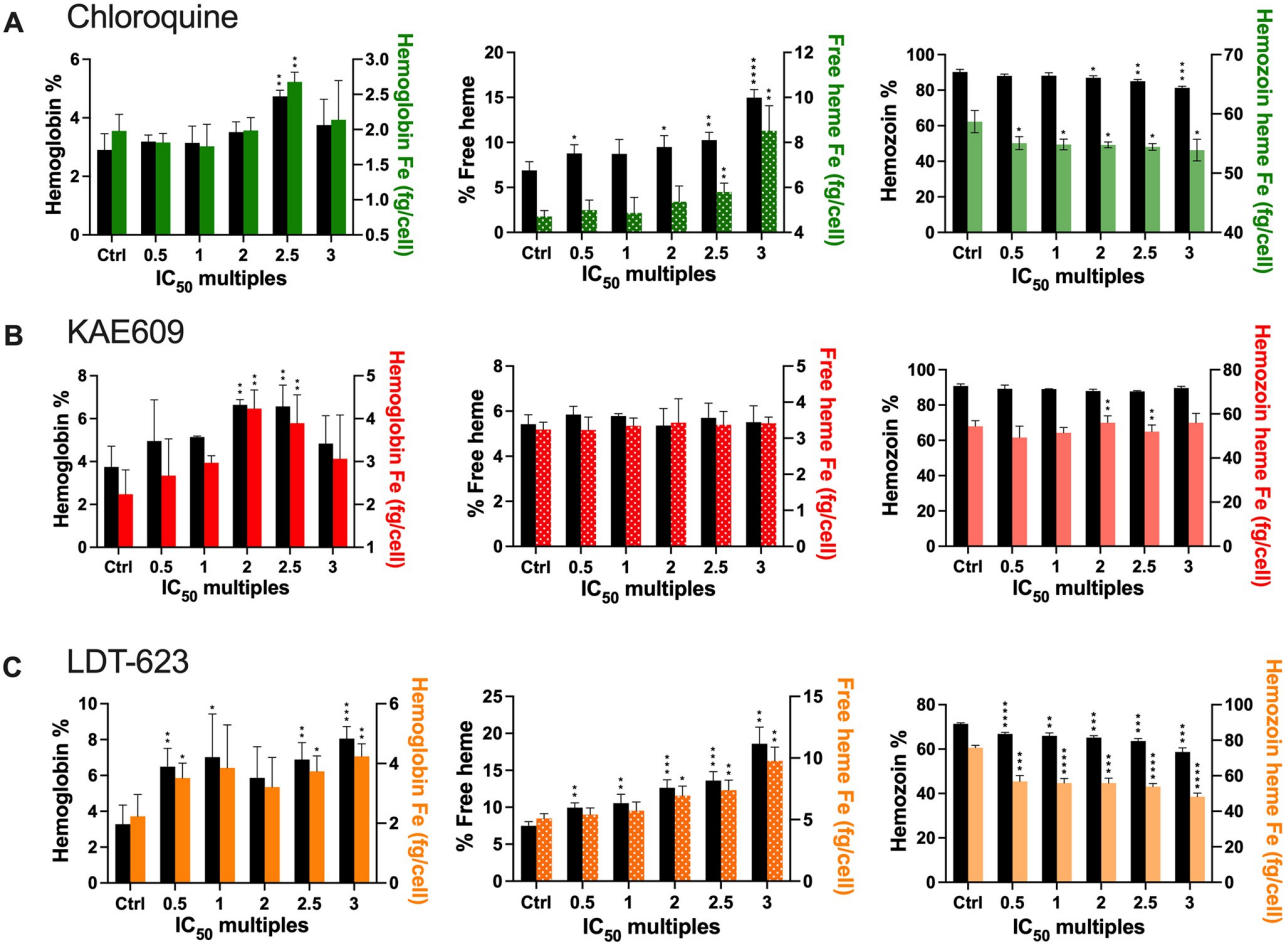
LDT-623 interferes with the hemozoin formation pathway within the digestive vacuole. (A) Parasites treated with LDT-623 display a more pronounced heme fractionation profile than CQ. (B) Heme fractionation profile of CQ-treated *P*. *falciparum* 3D7 parasites showing an increase in free heme and a decrease in Hz, as determined 32 h post-drug exposure. (C) KAE609 treatment did not interfere with heme or Hz accumulation. Percent levels of heme species (hemoglobin, free heme, or hemozoin) are represented by the black bars on the left y-axis, and absolute heme amounts (hemoglobin Fe, free heme Fe, or hemozoin heme Fe) determined from a heme standard curve and measured in femtograms per cell are represented in different colors (LDT-623 in red, CQ in orange, and KAE609 in green) on the right y-axis. Statistical comparisons of the drug-treated lines with their untreated controls were performed using two-tailed Student’s tests (with Welch’s correction). *p < 0.05; **p < 0.01; ***p < 0.001; ****p <0.0001.

Heme fractionation profiling of LDT-623 revealed a statistically significant increase in “free” heme in a concentration-dependent manner compared to untreated control (Fig 3A and S2 Table). Concurrently, we also observed significant concentration-dependent decreases in the levels of Hz. Moreover, an augmented percentage of undigested Hb was observed starting at 0.5 × IC_50_ of LDT-623. CQ, used as a positive control, also exhibited a significant increase in “free” heme starting at 0.5 × IC_50_ while Hz levels only decreased significantly from 2 × IC_50_. Interestingly, the proportion of “free” heme present as undigested Hb within the DV after incubation was significant at 2.5 × IC_50_ of CQ only (Fig 3B). As a negative control, parasites were treated with KAE609 (cipargamin), a PfATP4 inhibitor, which did not affect Hz formation [45]. Besides a subtle yet significant increment in Hb levels observed under 2 and 2.5 × IC_50_, no striking perturbations were noted for any of the heme species investigated under KAE609 treatment (Fig 3C). Altogether, these results demonstrate that LDT-623 interferes with the vital Hz formation pathway, presumably due to its quinoline core.

### 3.4. LDT-623 impacts PfCRT variant-specific drug efflux

PPQ and CQ resistance-conferring mutations in PfCRT allow positively charged drugs and other solutes to efflux into the cytosol [20,46]. Consequently, CQ- or PPQ-resistant parasites accumulate lower amounts of drugs within their DVs and prevent inhibition of heme detoxification. This contrasts with drug-sensitive parasites, where the increased intravacuolar concentrations of these drugs inhibit heme detoxification and result in subsequent parasite death.

**Fig 4:**
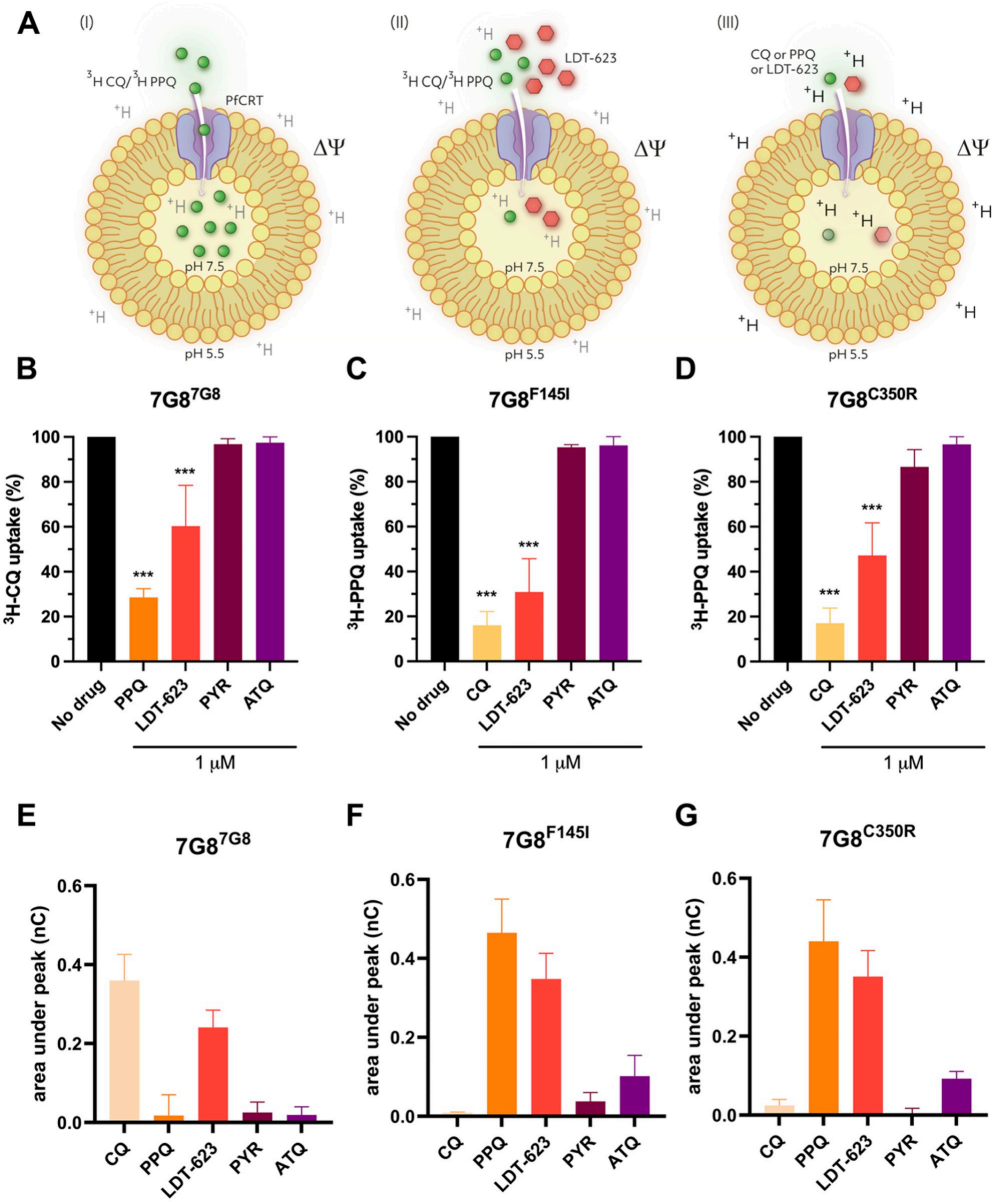
Uptake of the 4-AQs CQ, PPQ, and LDT-623 in PfCRT-containing proteoliposomes. (A) Schematic representation of the drug uptake assay highlighting the “inside-out” digestive vacuole aspect of PfCRT-containing proteoliposomes. Uptake of radiolabeled CQ or PPQ in the absence (I) or presence (II) of LDT-623 using the accumulation of radiolabeled CQ or PPQ as read-out or measuring 4-AQ transport-associated current fluxes (III) with electrophysiology. Uptake of 100 nM [^3^H]CQ (B) or [^3^H]PPQ (C-D) measured for 30 seconds in proteoliposomes containing the indicated PfCRT variants normalized to control liposomes. Data represent mean ± SEM of N = 3 independent experiments with n = 7 technical replicates each. (E-G) Transport of 10 μM CQ, PPQ, LDT-623, PYR, or ATQ by proteoliposomes containing indicated PfCRT variants using the SURFE^2^R N1-based SSM electrophysiological measurements. Data represent PfCRT isoform-specific currents (corrected for the non-specific signal measured in control liposomes lacking PfCRT) and are the mean ± SEM of N = 3 independent experiments with n = 6–9 technical replicates each.

Using PfCRT-containing proteoliposomes as a surrogate for drug transport, we employed an assay that mimics an “inside-out” parasite DV. This assay employed the CQ-resistant, PPQ-sensitive 7G8 PfCRT isoform, as well as two variants (7G8^F145I^ and 7G8^C350R^) that include an additional mutation that is observed in Southeast Asia and South America, respectively, and that mediate PPQ resistance and a reversion to CQ sensitivity [19,47]. Assays were designed to evaluate the impact of LDT-623 on PfCRT-mediated drug uptake in these 7G8 isoforms. The uptake of [^3^H]CQ or [^3^H]PPQ was measured in PfCRT-containing proteoliposomes using an internally directed pH gradient and ΔΨ (Fig 4A) and was normalized to that of control liposomes lacking PfCRT [20]. Drug-specific transport (here measured by [^3^H]-labeled drug accumulation) can be differentially mediated by distinct PfCRT isoforms, in agreement with varying resistance phenotypes as seen with the PfCRT isoforms used: 7G8^7G8^ (CQ-resistant, PPQ-sensitive), 7G8^F145I^ (CQ-sensitive, PPQ-resistant) and 7G8^C350R^ (CQ-sensitive, PPQ-resistant).

In the CQ-resistant 7G8 PfCRT isoform, the drug-free control showed that [^3^H]CQ uptake was maximal, as expected, based on its resistance phenotype (S3 Table). The addition of 1 μM LDT-623, however, reduced the uptake of [^3^H]CQ (to an average of 60.2% relative to [^3^H]CQ without another drug) (Fig 4B). As for the PPQ-resistant 7G8^F145I^ and 7G8^C350R^ isoforms, the addition of [^3^H]CQ also yielded a reduced percentage of [^3^H]PPQ uptake (30.% and 47.2%, respectively, relative to [^3^H]PPQ alone) (Fig 4C and 4D). No competitive inhibition of [^3^H]CQ or [^3^H]PPQ transport was observed with pyrimethamine (PYR) or atovaquone (ATQ), which have entirely distinct MoAs (inhibition of folate synthesis and the mitochondrial electron transport chain, respectively). Of note, [^3^H]CQ uptake by the PfCRT 7G8 isoform and [^3^H]PPQ uptake by the 7G8^F145I^ and 7G8^C350R^ isoforms were inhibited by the addition of the reciprocal agents PPQ and CQ (see Fig 4B–4D), to levels slightly greater than those observed with LDT-623. We interpret these data as evidence that PPQ and CQ can antagonize each other’s transport via appropriately mutated PfCRT variant isoforms. Earlier studies have shown that PPQ and CQ can bind drug-resistant and -sensitive PfCRT isoforms with similar K_d_ values, with the difference being that the resistant isoforms acquire transport properties [20].

Since both [^3^H]CQ and [^3^H]PPQ uptake are reduced in the presence of LDT-623 in the PfCRT isoforms tested herein, it can be speculated that LDT-623 competes with CQ and PPQ for transport by the distinct PfCRT isoforms. This notion is supported by the robustness of variant-specific 4-AQ interactions with PfCRT and by the minimal impact of ATQ- or PYR–cytochrome *bc1* or dihydrofolate reductase inhibitors, respectively [48,49], on the uptake of [^3^H]CQ or [^3^H]PPQ (Fig 4B–4D). To provide further experimental evidence for the interaction of LDT-623 with PfCRT, we established a robust platform to screen electrogenic events in the same PfCRT-containing proteoliposome-based model system using solid-supported membrane (SSM) electrophysiology [33,50]. This system enables the detection of ion fluxes associated with 4-AQ, e.g., CQ, PPQ, or LDT-623, transport. Our data (Fig 4E–4G) reveal that the addition of the three tested 4-AQs to the distinct PfCRT isoforms–as validated with parallel [^3^H]CQ and [^3^H]PPQ uptake studies (Fig 4B–4D)–elicited currents. We conclude that these currents, reflective of the change of the membrane potential in the PfCRT-containing proteoliposomes, are the result of 4-AQ transport-associated flux of protons (H^+^), according to an H^+^/4-AQ co-transport (symport) mechanism as we recently demonstrated [20].

### 3.5. Profiling of LDT-623 in *pfcrt* and *pfmdr1*-edited *P*. *falciparum* lines illustrate variant-mediated cross-resistance profiles

**Fig 5:**
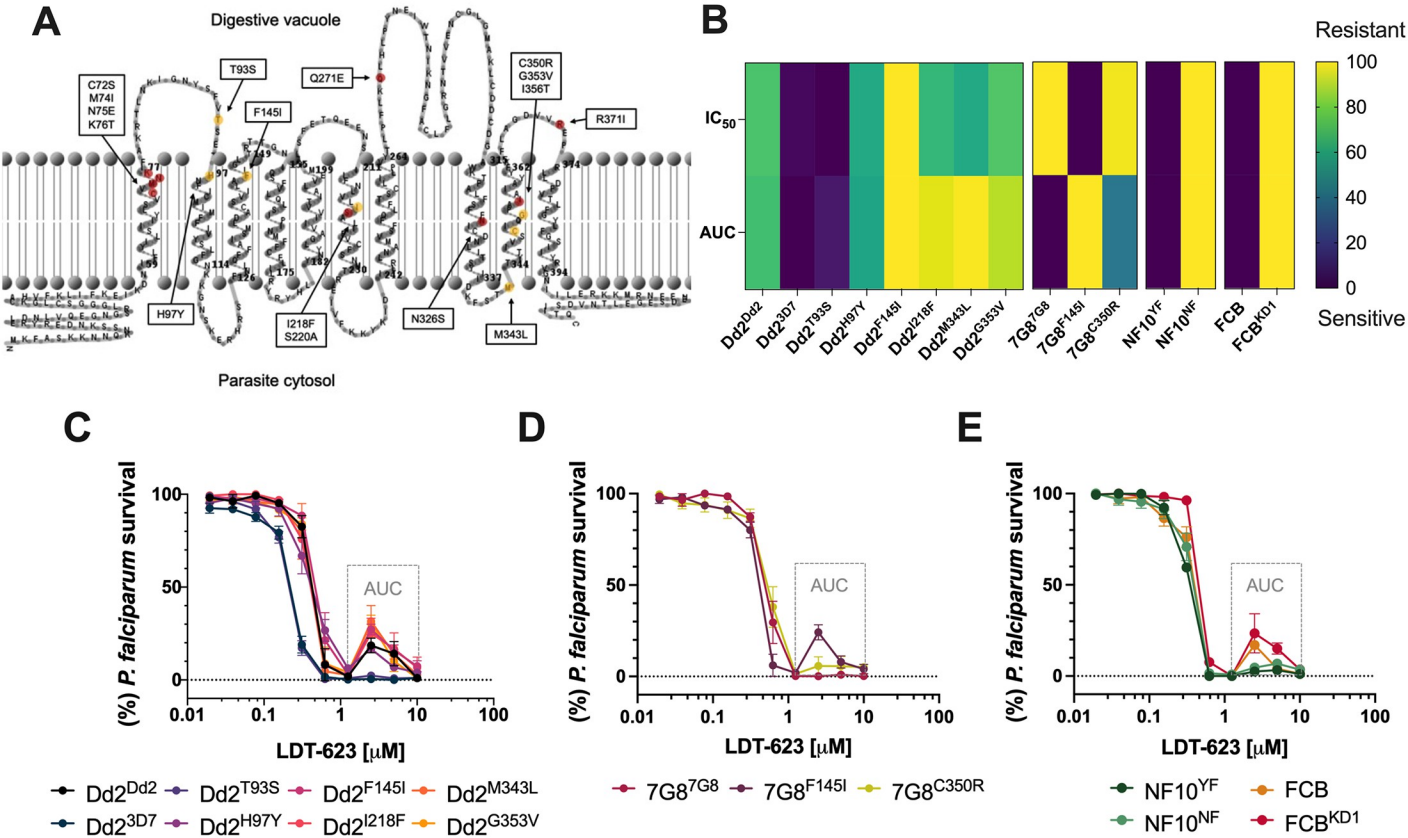
LDT-623 dose–response curves illustrate distinct variant PfCRT-mediated resistance profiles. (A) Mapping of CQ (red) and PPQ (yellow) resistance-conferring PfCRT amino acid substitutions on the 2D structure of PfCRT^3D7^ using TMRPres2D [59]. (B) Cross-resistance heatmap of IC_50_ and AUC values for LDT-623 across *pfcrt* and *pfmdr1*-edited *P*. *falciparum* lines (N = 2–4). Data were normalized across parameters and scaled into a 0–100 interval for heatmap coloring. (C-D) Dose-response curves for *pfcrt*-edited clones. Data show mean ± SEM percent parasite survival (relative to drug-free controls) as a function of LDT-623 concentration (N = 2–4). (D) Dose-response curves for *pfmdr1*-edited clones. Data show mean ± SEM percent parasite survival (relative to drug-free controls) as a function of LDT-623 concentration (N = 3).

To correlate drug uptake results with parasite susceptibility phenotypes, we used *pfcrt*-modified isogenic parasite lines previously generated [19,20,51–53] via zinc-finger nuclease editing (Table 1 and Fig 5A). We hypothesized that distinct PfCRT isoforms could display different binding transport activity patterns for 4-AQs, thereby modulating parasite susceptibility to LDT-623.

**Table 1:** Panel of *pfcrt-*edited *P*. *falciparum* lines used in this study.

Parasite line	Origin	Phenotype		PfCRT amino acid at listed positions															
		CQ	PPQ	72	74	75	76	93	97	145	218	220	271	326	343	350	353	356	371
Dd2^Dd2^	SE Asia	R	S	C	I	E	T	T	H	F	I	S	E	S	M	C	G	T	I
Dd2^T93S^	Cambodia	R	R	C	I	E	T	**S**	H	F	I	S	E	S	M	C	G	T	I
Dd2^H97Y^	Cambodia	R	R	C	I	E	T	T	**Y**	F	I	S	E	S	M	C	G	T	I
Dd2^F145I^	Cambodia	S ^a^	R	C	I	E	T	T	H	**I**	I	S	E	S	M	C	G	T	I
Dd2^I218F^	Cambodia	S	R	C	I	E	T	T	H	F	**F**	S	E	S	M	C	G	T	I
Dd2^M343L^	Cambodia	R	R	C	I	E	T	T	H	F	I	S	E	S	**L**	**C**	G	T	I
Dd2^G353V^	Cambodia	S	R	C	I	E	T	T	H	F	I	S	E	S	M	C	**V**	T	I
Dd2^3D7^	Africa	S	S	C	M	N	K	T	H	F	I	A	Q	N	M	C	G	I	R
7G8^7G8^	S. America, W. Pacific	R	S	S	M	N	T	T	H	F	I	S	Q	D	M	C	G	L	R
7G8^F145I^	Experimental	S	R	S	M	N	T	T	H	**I**	I	S	Q	D	M	C	G	L	R
7G8^C350R^	French Guiana	S	R	S	M	N	T	T	H	F	I	S	Q	D	M	**R**	G	L	R

^a^ Sensitized to CQ compared to the parental line but not fully sensitive. *pfcrt* editing by customized zinc-finger nucleases was confirmed using Sanger sequencing. Differences from the 3D7 wild-type allele are shown in gray shading for both Dd2 and 7G8. Bold indicates mutations identified from piperaquine-resistant field isolates. S: sensitive, R: resistant. Table adapted from [19,20,51].

PfMDR1, another DV-localized transporter, is a major determinant of pleiotropic antimalarial resistance in the field. Studies have shown that changes in both copy number variations and amino acids at position 86 in *pfmdr1* significantly impact parasite susceptibility to several antimalarial drugs [54,55]. Because there appears to be an interplay between specific *pfcrt* and *pfmdr1* alleles [56,57], we also explored the role of genetically manipulated *pfmdr1* on the susceptibility of *P*. *falciparum* to LDT-623. We used the NF10^YF^ clone (progeny of a genetic cross between 7G8, from Brazil, and GB4 parasites, from Gabon) that harbors a single copy of the GB4 *pfmdr1* allele encoding the N86Y/Y184F (YF) haplotype, and the NF10^NF^ clone, genetically engineered to express the N86/Y184F (NF) haplotype [55]. Additionally, we assessed the impact of *pfmdr1* amplification on the activity of LDT-623 using the parental line FCB and FCB^KD1^ that express two or one copies of *pfmdr1*, respectively [54].

Most of the *pfcrt*-edited *P*. *falciparum* lines assessed did not show substantial rightward shifts in their survival curves (Fig 5C–5E). For comparison, previously published CQ and PPQ susceptibility data for the parasite strains tested were listed alongside LDT-623 results (S4 and S5 Tables). Even though initial LDT-623 concentrations similarly killed different parasite lines, specific sets of mutations differentially impacted parasite survival under higher drug concentrations, resulting in incomplete killing. This incomplete killing phenotype, characterized by biphasic dose-response curves and associated with PPQ resistance [19,58], reveals atypical profiles for typical 72 h dose-response assays, complicating the IC_90_ determination. In this way, AUC was calculated within the region of the survival curve limited by drug concentrations from 1.25 to 10 μM, encompassing a range of biphasic responses. Lower, yet not statistically significant, AUC for Dd2^3D7^ and Dd2^T93S^ (*p*-value: 0.1, Mann-Whitney U test) suggest an increase in susceptibility to LDT-623 compared to the isogenic Dd2^Dd2^ (Fig 5B). 7G8 clones 7G8^F145I^ and 7G8^C350R^, linked to PPQ resistance, were the closest to statistical significance in the AUC analysis compared to the isogenic 7G8^7G8^ (*p*-value: 0.06; Mann-Whitney U test), suggesting a trend towards diminished susceptibility to LDT-623 (Fig 5B). Moreover, the PfCRT F145I mutation seems to play a role in resistance to high concentrations of LDT-623 by protecting parasites via an incomplete killing phenotype, regardless of the parasite’s genetic background.

Regarding *pfmdr1* genetic changes, the N-terminal N86 mutation in NF10^NF^ did not show any impact on the activity of LDT-623 compared to its parent NF10^YF^ at the IC_50_ level (1.1-fold increase, *p*-value: 0.7, Mann-Whitney U-test). While analyzing *pfmdr1* copies, the knockdown clone FCB^KD1^ showed a 1.2-fold increase in IC_50_ compared to FCB (Fig 5B), a behavior like that previously observed for CQ [54]. However, this increase was not statistically significant (*p*-value: 0.1, Mann-Whitney U-test). Finally, the knockdown FCB^KD1^ displayed a more pronounced biphasic curve compared to its FCB parent but no statistical significance was achieved between their AUC values (*p*-value: 0.4; Mann-Whitney U test). Overall, even though there appears to be a tendency of slight resistance regarding a few of the lines assayed, no striking differences in parasite susceptibility via AUC analysis were observed. None of the *pfcrt* or *pfmdr1* relevant field mutations engineered into these parasite lines yielded significant cross-resistance.

### 3.6. LDT-623 is refractory to resistance selection *in vitro*

**Fig 6:**
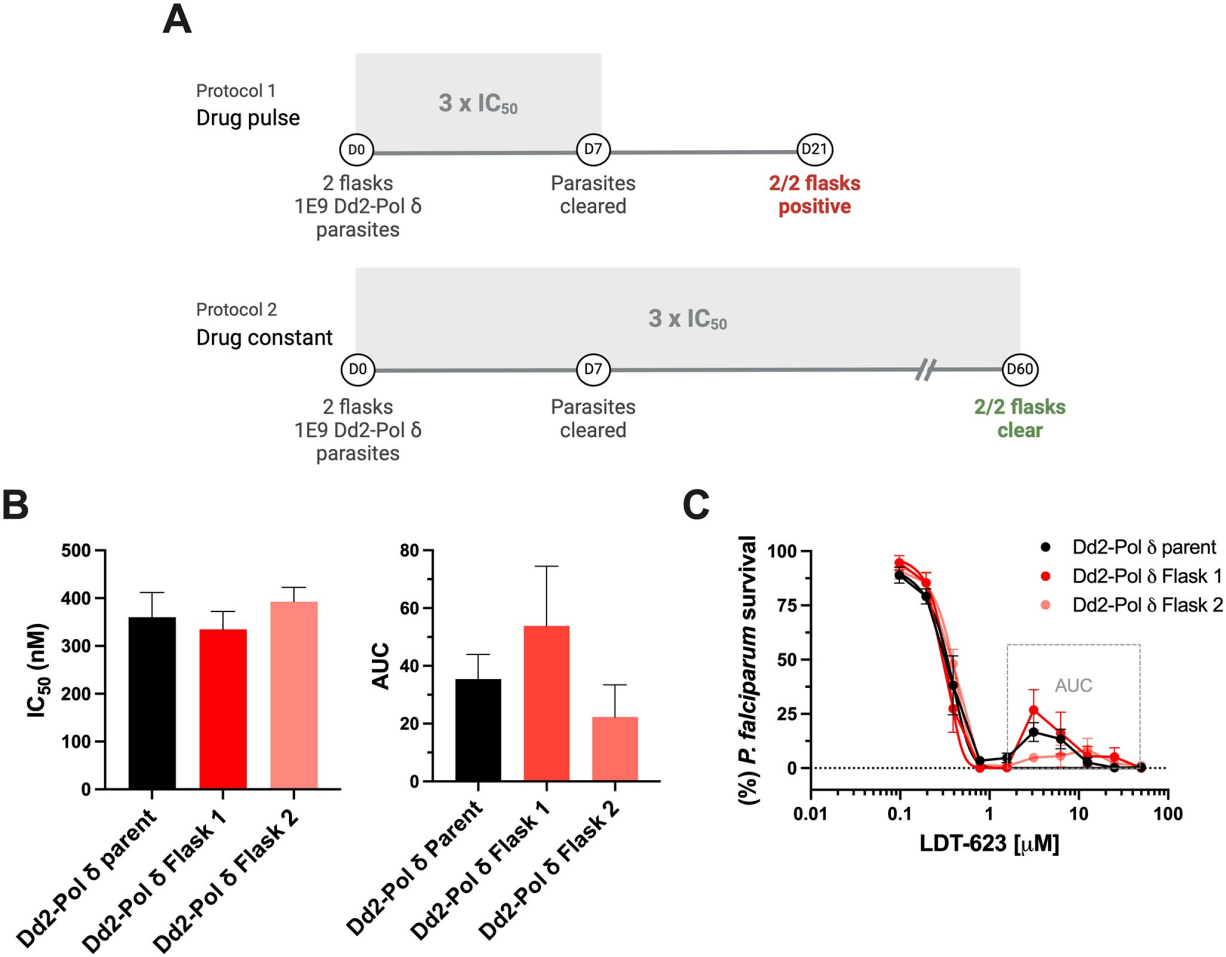
LDT-623 does not readily select for resistance *in vitro*. (A) Selection of resistance to LDT-623 using the Dd2-Pol*δ* line. (A) Parasites were cultured in the presence of 1 μM LDT-623 (~3 × IC_50_). Parasite inoculum was set at 1 × 10^9^ in duplicate flasks. Shading indicated the presence of drug pressure in the protocol. (B) LDT-623 IC_50_ and AUC values for parental and recrudescent Dd2-Polδ lines under LDT-623 pressure. (C) Dose-response curves of LDT-623 for parental line (Dd2-Polδ) *versus* drug-pressured lines (Dd2-Polδ Flask 1 and Dd2-Polδ Flask 2). Arrow represents the 3 × IC_50_ concentration used for resistance selection. Data represent mean ± SEM (N = 5). Statistical significance was determined by Mann-Whitney U tests, *p<0.05.

In a bid to obtain additional insights into the potential molecular targets or pathways that drive the multistage antiplasmodial activity of LDT-623, we carried out two parallel resistance selection protocols to generate drug-resistant parasites (Fig 6A).

No parasite recrudescence was observed in the set-up where constant pressure was applied throughout the 60 days of selection. In the set-up in which drug pressure was removed after day 7 following parasite clearance, recrudescence was observed in both flasks on day 21. However, drug response assays showed no difference in IC_50_ between the Dd2-Polδ parent culture and the recrudescent bulk parasites from either flask (Fig 6B). Given the biphasic shape of the parasite survival curves, an AUC analysis was performed. While the mean AUC for the Dd2-Polδ parent culture was 35.4, parasites recovered in Flasks 1 and 2 had values of 53.8 and 22.3, respectively (*p*-values: 0.7 and 0.3, Mann-Whitney U test; Fig 6B), revealing no significant difference between the curves (Fig 6C and S6 Table). With no LDT-623-resistant parasites recovered under constant drug pressure, the log minimum inoculum of resistance (MIR) calculated for LDT-623 is higher than 9 (i.e. >10^9^ infected RBCs), providing evidence for a robust barrier for resistance.

### 3.7. Failure to recover parasites in the resistome assay indicates no cross-resistance of LDT-623 with major targets and resistance mechanisms

Cross-resistance profiling of compounds against parasite lines with known resistance mutations is another approach to gain insight into the potential resistance liabilities of a compound. Leveraging drug-resistant lines generated predominantly by the MalDA consortium using *in vitro* evolution or genetic engineering has aided the composition of a library of >50 parasites that, altogether, cover a broad range of currently known resistance mechanisms as well as notable targets advancing in the contemporary antimalarial drug discovery pipeline [60–62]. CRISPR-based barcode insertion allowed these representative parasite lines to be pooled and co-cultured, with their relative abundances quantified by barcode sequencing (S7 Table) [40]. Using a multiplexed competitive growth assay, we subjected this pool of barcoded parasites to LDT-623 pressure, as a means to identify potential cross-resistance targets.

Selective pressure of 3 × IC_50_ LDT-623 for Dd2 (~790 nM) was applied to the pool of barcoded parasites and maintained constant throughout the assay. At the same time, cumulative growth profiles were recorded for LDT-623, KDU691 – a known PI4-kinase inhibitor [63], and an untreated control (S3 Fig). After 14 days of incubation, no growth recovery was observed for the pool of barcoded parasites treated with LDT-623 and thus no barcodes could be detected by PCR. In contrast, treatment of the pool with the PI4-kinase inhibitor KDU691 yielded recrudescence after 9 days and enrichment of barcode profiles of PI4-kinase mutant parasites (S3B Fig). Failure of these parasite lines to grow under LDT-623 pressure is a strong indicator of no cross-resistance with major targets and resistance mechanisms evaluated, such as ABC transporter I (ABCI3), P-type ATPase (ATP4), cyclic amine resistance locus (CARL), and translation elongation factor 2 (eEF2) [64], among others [62] (S7 Table).

## 4. Discussion and conclusions

Even though 4-AQs have been relatively well-explored, the history of drug development endorses their adoption as partner drugs in antimalarial therapy, as exemplified by amodiaquine, PPQ, and pyronaridine. Based on a previously characterized antiplasmodial molecule [23], a similarity screening coupled with *in vitro* assays identified LDT-623, a 4-AQ with nanomolar ABS activity. Previously described as an ABS inhibitor in high throughput screening endeavors [65], we aimed to explore this molecule’s potential by demonstrating its hepatic schizonticidal and transmission-blocking activity in *P*. *falciparum* mature gametocytes and *P*. *berghei* ookinetes. This moderate yet unusual multistage activity that distinguishes LDT-623 from the typical 4-AQ phenotype encouraged us to investigate it further.

Using a cellular heme fractionation assay, we demonstrated that LDT-623 interferes with the heme detoxification pathway within the DV in a concentration-dependent manner. This can be linked to its MoA. Numerous point mutations in *pfcrt* have been proven to mediate CQ [18] and PPQ resistance [66] to varying extents. These mutation-driven phenotypes support parasite evasion from the inhibition of Hz formation within the DV: outward drug transport is significantly greater in PfCRT-mutated forms, leading to a lesser extent of drug accumulation in its site of action, thus rendering the parasite resistant. To further dissect the contribution of these complimentary mechanisms to the MoA of LDT-623, we resorted to isoforms of PfCRT-reconstituted proteoliposomes [20]. Measurement of the active uptake of radiolabeled CQ and PPQ showed that LDT-623 partially interferes with drug transport by PfCRT, suggesting that LDT-623 can compete with the other 4-AQs for their interaction with the same functional site. Indeed, electrophysiological measurements support this notion, as LDT-623 elicits the same magnitude of currents as CQ and PPQ. These currents are considered to reflect PfCRT-mediated H^+^-coupled 4-AQ symport, according to which the transport of 4-AQ compounds is associated with the flux of H^+^ in the same direction [20].

Functionally, differential drug transport affinities between these PfCRT variants might mirror distinct drug sensitivity profiles. To this end, we put together a panel of *pfcrt*-edited *P*. *falciparum* lines. To evaluate the potential cross-resistance of LDT-623 with globally relevant *pfcrt* mutations, we selected not only those haplotypes mutated at positions 72–76 from Southeast Asia/Asia (CVIET) and South America (SVMNT) that drive CQ resistance [52] but also PPQ-resistance-conferring mutations such as H97Y, F145I, M343L, or G353V, observed in Southeast Asia [19], and the C350R mutation that has been recently shown to impact over 2/3 of parasite populations analyzed in the Guianas [67–69]. Most Dd2 lines assayed, except for Dd2^3D7^ (*wild-type* PfCRT) and Dd2^T93S^, show a biphasic response at high LDT-623 concentrations. This same phenotype is not observed in the 7G8^7G8^ line with the South American SVMNT PfCRT isoform. We believe that CVIET PfCRT could be the major driver of these bimodal dose-response curves. Overall, we hypothesize that a gain of compound transport by mutant *pfcrt* might be the underlying reason that LDT-623 loses some activity in a range of higher concentrations, as also seen by increased PPQ transport with the highly PPQ-resistant Dd2^F145^ and Dd2^T93S^ parasite lines [20,52]. Although LDT-623 activity hints towards diminished potency against F145I, data is not a proxy for potential clinical settings, as this mutation is highly unfit in the Dd2 background [19] and no reports have shown its emergence in 7G8 parasites.

Because there appears to be an interplay between particular *pfcrt* and *pfmdr1* alleles [56,57], we explored the impact of *pfmdr1*—a marker of resistance to lumefantrine and mefloquine (partner drugs in ACTs), on activity of LDT-623. The FCB line, besides its CVIET PfCRT haplotype, also harbors the *pfmdr1* N86Y mutation (cognate to NF10^YF^, yet in a distinct genetic background). Interestingly, although NF10 are also CVIET-bearing parasites, they seem to preserve the sigmoidal curve shape—as opposed to the biphasic response from FCB parasites. Revisiting the trend observed for Dd2 regarding the bimodal curve believed to be driven by CVIET, in this case, we believe that the incomplete killing for both FCB and FCB^KD1^ might be due to either a strain-specific behavior or even to possible specific interactions between these *pfcrt* and *pfmdr1* alleles. A few of the mutant PfCRT and PfMDR1 haplotypes evaluated in this study also play a role in the parasite susceptibility to other antimalarial drugs, as seen with increased lumefantrine susceptibility and sensitivity to ART derivatives [70–72]. Additionally, increased *pfmdr1* copy numbers are commonly observed in Southeast Asia [54,72]. These amplified *pfmdr1* copies, fortunately, do not usually extend to African parasite populations, where the fitness costs potentially do not support their expansion in highly endemic areas [73]. Given that we found no pre-existing cross-resistance with *pfcrt-* or *pfmdr1*-mutated parasites, the results brought by the present study are timely within the context of considering 4-AQs as effective partner drugs. Our finding that inhibition of Hz formation is part of the MoA of LDT-623, along with the absence of the heme detoxification pathway in both liver and insect parasite stages [13] implies a multifaceted MoA, whose basis requires further investigation. Exploring mechanisms of antimalarial compounds beyond ABS is notoriously difficult given the lower parasite loads available in parasite hepatic and sexual stages compared to ABS. Similarly, insect stages are of extremely low density in the mosquito midgut.

We then resorted to hypermutable Dd2-Pol δ ABS parasites that can display an increased mutation rate of up to 28-fold under drug pressure [38], hence expanding the genetic diversity available for the parasite populations during the selective pressure and increasing our chances of identifying a potential separate MoA of LDT-623. Even though this parasite line does not represent a direct proxy for field outcomes, it is a reliable tool to gain further insights into a drug’s MoA. This strategy has been successful in generating mutants for antimalarial compounds dubbed “irresistible” [38,74], including ones that have previously failed to select for mutations in 3D7 and Dd2 parasites. The failure of LDT-623 to select resistant parasites *in vitro* suggests that this event might be unlikely to occur in the field. Among numerous potential reasons for this compound not to select for resistance, the bimodal phenotype for LDT-623 recrudescent parasites may reveal a subset of parasites surviving high drug concentrations. This biphasic response could be linked to concentration-dependent activation of drug efflux mechanisms [16,75,76] or even a polypharmacological trait translating into multiple MoAs of this compound [75], which corroborates the multistage antiplasmodial activity observed for LDT-623. Moreover, considering the absence of evidence of lumefantrine and pyronaridine resistance in *P*. *falciparum* strains to date and the fact that amodiaquine remains widely effective [77], the study is timely when contemplating 4-AQs as effective partner drugs. With ferroquine undergoing phase 2 trials as a first-line partner drug [72,78–82], including in combination with OZ439, revisiting 4-AQs is appealing. In light of this, a novel 4-AQ antimalarial, such as LDT-623, showing no cross-resistance to these relevant genetic changes, may constitute a valuable tool for further drug optimization efforts.

Finally, we explored parasite modulators of LDT-623 susceptibility and gain insight into its MoA using a fitness competition assay employed to identify cross-resistant barcoded parasite lines able to outgrow drug pressure [40]. Strikingly, none of the parasite strains assayed were retrieved. It is also important to mention that compounds like LDT-623, which not only seem to possess a multifaceted MoA but also leverage host-specific processes (such as the heme detoxification pathway) are compelling tools that have the potential to make it more challenging for resistance to evolve [83,84]. Altogether, by leveraging LDT-623’s ability to bypass drug efflux mechanisms in mutant PfCRT and PfMDR1, two major resistance determinants in ACT partner drugs, our data encourage revisiting quinolines as components of malaria treatment strategies such as triple ACTs, as demonstrated for other antimalarial combinations [19,51].

## Supporting information

S1 Fig **(A)** Structure of compound LDT-611. **(B)** *In vitro* growth inhibition of chloroquine-sensitive and resistant *P*. *falciparum* strains for LDT-611 and chloroquine, an antimalarial standard (data are represented as mean ± SD, N = 3). **(C)** Virtual screening workflow for the identification of LDT-611 structural analog molecules. **(D)** Radial plot showing structural similarity between LDT-611 and its structural analogs selected with Tanimoto coefficient > 0.78.
(PNG)

S2 Fig Activity of chloroquine on *P*. *berghei* (ookluc) gamete fertilization and consequent ookinete formation on conversion assay. Luciferase activity (light emission in relative light units) is expressed as means ± SEM and is directly proportional to gamete fertilization and ookinete conversion. (N = 2–3).
(PNG)

S3 Fig Resistome assay establishes no cross-resistance between LDT-623 and major drug targets and mechanisms of antimalarial resistance. **(A)** Cumulative growth profiles for LDT-623 (4-AQ), a known PI4-kinase inhibitor KDU691, and an untreated control. Drug pressure of 3 x IC_50_ was maintained constant for 14 days and the pool exhibited no growth recovery for LDT-623, whereas recrudescence was observed after 7 days with KDU691. **(B)** Barcode profiles (days 0 and 14) for the no-drug control and the recovered parasites treated with KDU691. Cultures treated with KDU691 showed enrichment of PI4-kinase mutant parasites. Cultures were run in triplicate.
(JPG)

S1 Table Data supporting the multistage antiplasmodial activity of LDT-623 (*P*. *falciparum* stage VI-V gametocytes, *P*. *berghei* ookinetes, and *P*. *berghei* liver schizonts). (XLSX)

S2 Table Absolute values of heme species (Hb, Heme, Hz) identified upon heme fractionation profiling of LDT-623, chloroquine, and KAE609. (XLSX)

S3 Table Data on transport assays using PfCRT-containing proteoliposomes. (XLSX)

S4 Table Antimalarial IC_50_ and IC_90_ values of LDT-623 tested in *pfcrt*-edited parasite lines. (XLSX)

S5 Table Antimalarial IC_50_ and IC_90_ values of LDT-623 tested in *pfmdr1*-modified parasite lines. (XLSX)

S6 Table *In vitro* survival curves of *P*. *falciparum* populations after LDT-623 resistance selection. (XLSX)

S7 Table Starting composition (% proportion on day 0) of each barcoded line for the resistome assay. Strain background (3D7 or Dd2), gene name and ID, mutation, and input % are indicated. Growth of parasite pool is shown in S3 Fig.
(XLSX)
