# Supplementary figures and images for: A novel 4-aminoquinoline chemotype with multistage antimalarial activity and lack of cross-resistance with PfCRT and PfMDR1 mutants
Source: PLoS Pathog. 2024 Oct 29;20(10):e1012627. doi: 10.1371/journal.ppat.1012627 (PMC11521309; doi:10.1371/journal.ppat.1012627)

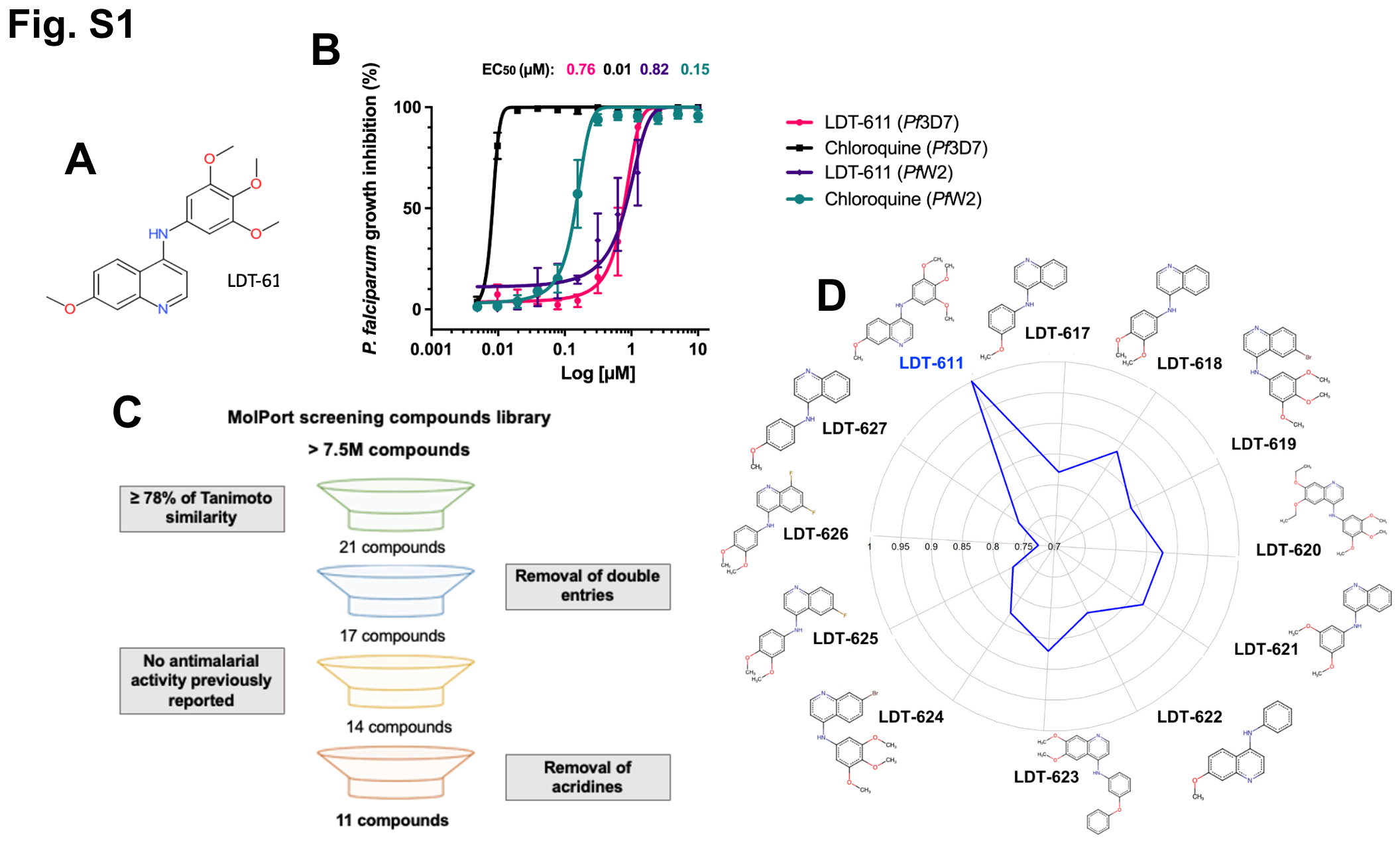

Supplement: S1 Fig — (A) Structure of compound LDT-611. (B) In vitro growth inhibition of chloroquine-sensitive and resistant P. falciparum strains for LDT-611 and chloroquine, an antimalarial standard (data are represented as mean ± SD, N = 3). (C) Virtual screening workflow for the identification of LDT-611 structural analog molecules. (D) Radial plot showing structural similarity between LDT-611 and its structural analogs selected with Tanimoto coefficient > 0.78. (PNG) [file ppat.1012627.s001.png]

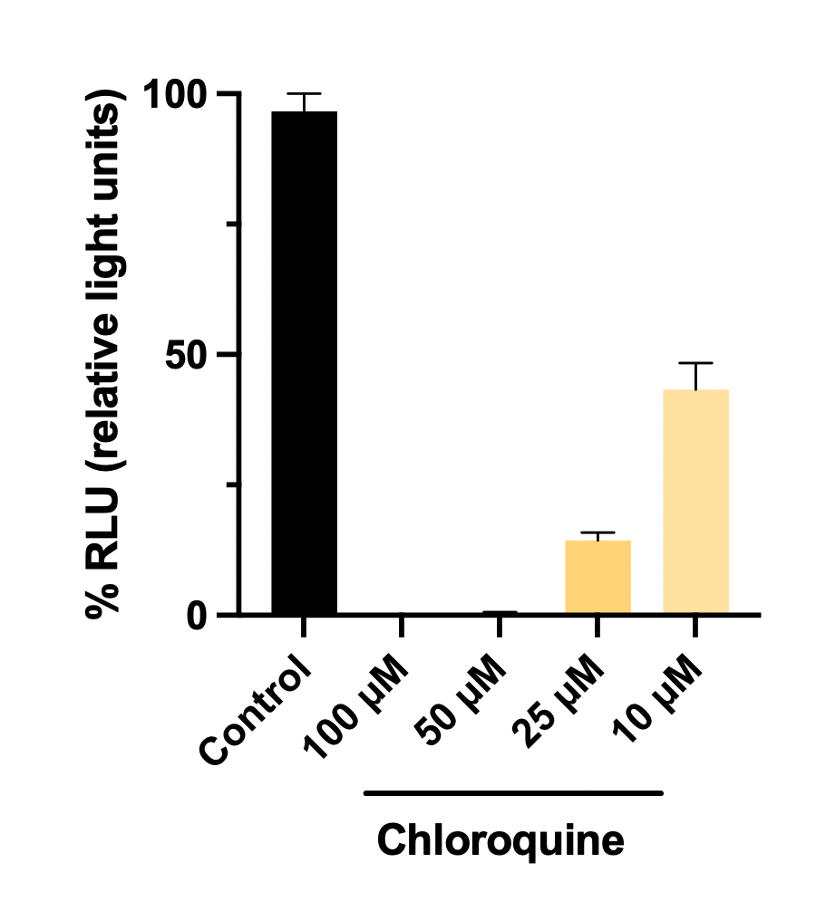

Supplement: S2 Fig — Luciferase activity (light emission in relative light units) is expressed as means ± SEM and is directly proportional to gamete fertilization and ookinete conversion. (N = 2–3). (PNG) [file ppat.1012627.s002.png]

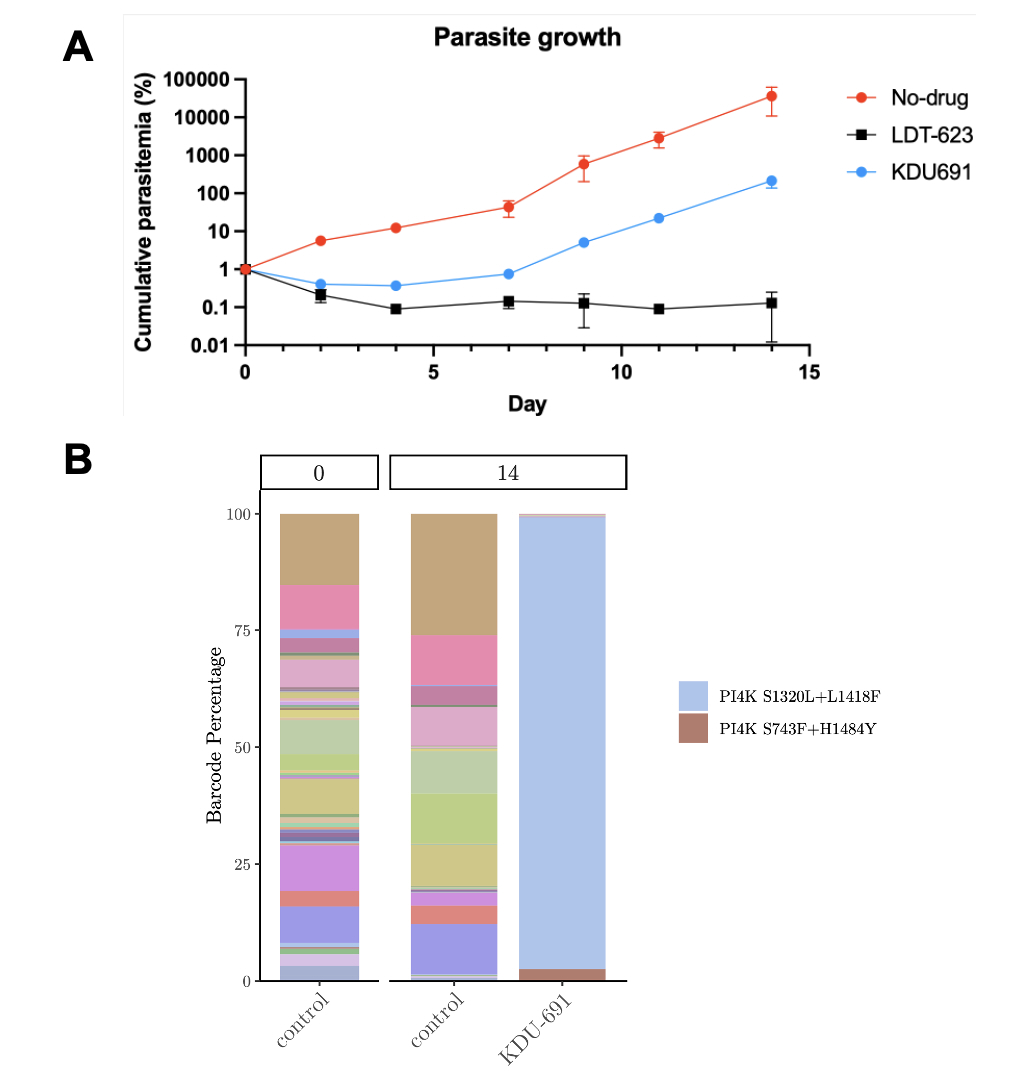

Supplement: S3 Fig — (A) Cumulative growth profiles for LDT-623 (4-AQ), a known PI4-kinase inhibitor KDU691, and an untreated control. Drug pressure of 3 x IC50 was maintained constant for 14 days and the pool exhibited no growth recovery for LDT-623, whereas recrudescence was observed after 7 days with KDU691. (B) Barcode profiles (days 0 and 14) for the no-drug control and the recovered parasites treated with KDU691. Cultures treated with KDU691 showed enrichment of PI4-kinase mutant parasites. Cultures were run in triplicate. (JPG) [file ppat.1012627.s003.jpg]
